# Lipid Chaperones and Metabolic Inflammation

**DOI:** 10.4061/2011/642612

**Published:** 2011-10-30

**Authors:** Masato Furuhashi, Shutaro Ishimura, Hideki Ota, Tetsuji Miura

**Affiliations:** Second Department of Internal Medicine, Sapporo Medical University School of Medicine, S-1, W-16, Chuo-ku, Sapporo 060-8543, Japan

## Abstract

Over the past decade, a large body of evidence has emerged demonstrating an integration of metabolic and immune response pathways. It is now clear that obesity and associated disorders such as insulin resistance and type 2 diabetes are associated with a metabolically driven, low-grade, chronic inflammatory state, referred to as “metaflammation.” Several inflammatory cytokines as well as lipids and metabolic stress pathways can activate metaflammation, which targets metabolically critical organs and tissues including adipocytes and macrophages to adversely affect systemic homeostasis. On the other hand, inside the cell, fatty acid-binding proteins (FABPs), a family of lipid chaperones, as well as endoplasmic reticulum (ER) stress, and reactive oxygen species derived from mitochondria play significant roles in promotion of metabolically triggered inflammation. Here, we discuss the molecular and cellular basis of the roles of FABPs, especially FABP4 and FABP5, in metaflammation and related diseases including obesity, diabetes, and atherosclerosis.

## 1. Introduction

Inflammation is classically characterized as heat (calor), pain (dolor), redness (rubor), and swelling (tumor) [1]. The short-term adaptive response of inflammation is crucial for integration of injury response and repair in cells and tissues. However, the long-term consequences of prolonged inflammation are often not beneficial. It has recently been shown that low-grade and chronic features of inflammation are observed in metabolic diseases including obesity, insulin resistance, type 2 diabetes, and cardiovascular disease [2, 3]. This atypical immune response emerging from metabolic tissues is referred to as metabolically triggered inflammation, “metaflammation,” which is principally triggered by nutrients and metabolic surplus, resulting in the engagement of at least a subset of molecules and signaling pathways involved in classical and canonical inflammation [2].

A number of hormones, cytokines, and bioactive lipids function in both metabolic and immune responses. Metabolic and immune systems regulate each other by the same cellular machinery. In metabolically active cells such as adipocytes and macrophages, metaflammatory pathways can be initiated by not only extracellular mediators such as cytokines and lipids, particularly saturated fatty acids, but also by intracellular stresses such as endoplasmic reticulum stress and excess production of reactive oxygen species derived from mitochondria. Signals from all of these mediators converge on inflammatory signaling pathways, including signaling kinases: c-Jun N-terminal kinase (JNK), inhibitor of nuclear kappa B kinase (IKK), protein kinase R (PKR), and others. These pathways lead to the inhibition of insulin signaling [4–6] and a vicious spiral of additional production of inflammatory mediators through transcriptional regulation using activating protein-1 (AP-1) and nuclear factor-kappa B (NF-*κ*B).

In this paper, we will focus on metabolically active cell-derived fatty acid-binding proteins (FABPs), which have been shown to regulate inflammatory and metabolic responses mainly in adipocytes and macrophages, and also discuss molecular and cellular links between FABPs and metaflammation, particularly in the context of metabolic diseases such as obesity, diabetes, and atherosclerosis.

## 2. Fatty Acid-Binding Protein (FABP) as a Lipid Chaperone

FABPs are a family of 14-15-kDa proteins that coordinate lipid trafficking and responses in cells [7]. FABPs can reversibly bind to hydrophobic ligands, such as saturated and unsaturated long-chain fatty acids, eicosanoids, and other lipids, with high affinity and broad selectivity. To date, at least 9 different FABP isoforms have been identified. Different members of the FABP family are expressed most abundantly in tissues involved in active lipid metabolism. The family contains liver (L-FABP/FABP1), intestinal (I-FABP/FABP2), heart (H-FABP/FABP3), adipocyte (A-FABP/FABP4/aP2), epidermal (E-FABP/FABP5/mal1), ileal (Il-FABP/FABP6), brain (B-FABP/FABP7), myelin (M-FABP/FABP8), and testis (T-FABP/FABP9) isoforms. FABPs have been proposed to facilitate the transport of lipids to specific compartments in the cell, such as to the mitochondrion or peroxisome for oxidation, to the nucleus for lipid-mediated transcriptional regulation, to the endoplasmic reticulum for signaling, trafficking, and membrane synthesis, to cytoplasmic enzymes for activity regulation, and to the cytoplasm for storage as lipid droplets. However, regulatory mechanisms of tissue-specific expression and function of various FABPs are still poorly understood. Specific contribution of each type of FABP to cell biology, physiology, and lipid metabolism had not been demonstrated until FABP-deficient mice models were created.

## 3. Adipocyte/Macrophage FABPs

Among the FABPs, FABP4, known as adipocyte FABP (A-FABP) or adipocyte P2 (aP2), is one of best-characterized isoforms (Table 1). FABP4 is highly expressed in adipocytes, making up about 1% of all soluble proteins in adipose tissue [8]. FABP5, another FABP known as epidermal FABP (E-FABP) or mal1, is expressed most abundantly in epidermal cells of the skin but is also present in several other tissues and cells including adipocytes [7] (Table 1). FABP5 constitutes a minor fraction of FABPs in adipocytes, the amount being about 100-fold smaller than that of FABP4 in adipocytes [9]. These two proteins, FABP4 and FABP5, have 52% amino acid similarity and bind to a variety of fatty acids with similar selectivity and affinity [10]. Interestingly, both FABP4 and FABP5 are also expressed in macrophages and dendritic cells [11, 12]. The stochiometry of these two proteins appears to be approximately equal in macrophages under physiological conditions [11]. The content of FABP4 in adipocytes is about 10,000-fold higher than that in macrophages [13]. In a state of germline FABP4 deficiency, FABP5 expression exhibits a strong compensatory increase in adipose tissue but not in macrophages or dendritic cells [11, 12, 14]. It has been demonstrated that both FABP4 and FABP5 play important roles in the regulation of insulin sensitivity and the development of atherosclerosis and that their impacts differentially involve adipocytes or macrophages [11, 14–22].

### 3.1. FABP4 (A-FABP/aP2)

Expression of FABP4 in adipocytes is highly regulated during differentiation of adipocytes and is transcriptionally controlled by fatty acids, peroxisome proliferator-activated receptor (PPAR) *γ* agonists, dexamethasone, and insulin [23–27]. Potential functional domains of FABP4 have been reported to include a nuclear localization signal, its regulation site, and a nuclear export signal [7, 62, 63]. The primary sequence of FABP4 does not demonstrate a readily identifiable nuclear localization signal or nuclear export signal. However, the signals could be found in the tertiary structure of FABP4. It has also been shown that there is a protein-protein interaction between FABP4 and hormone-sensitive lipase [28]. In this model, it has been postulated that FABP4 binds to and activates hormone-sensitive lipase in adipocytes, resulting in regulation of lipolysis. Adipocytes in FABP4-deficient mice exhibited reduced efficiency of lipolysis [29, 30]. Interestingly, during experimentally induced lipolysis, FABP4-deficient mice also revealed reduction in insulin secretion [29]. As another protein-protein interaction, ligand-bound FABP4 has been reported to bind to Janus kinase 2 (JAK2) and attenuate its signaling, indicating a new role for FABP4 as a fatty acid sensor affecting cellular metabolism [31]. It has also been reported that phosphatase and tensin homolog on chromosome 10 (PTEN), which negatively regulates the phosphoinositide 3-kinase pathway, interacts with FABP4, possibly regulating of lipid metabolism and adipocyte differentiation [32]. Interestingly, PTEN-null keratinocytes showed an elevated expression of FABP4, suggesting that PTEN plays a role in the transcriptional regulation of FABP4 expression [55].

In the whole body metabolic phenotype, FABP4-deficient mice exhibited an increase in body weight but reduced insulin resistance in the context of both dietary and genetic obesity [14, 15]. RNA interference-mediated *Fabp4* germline knockdown in mice on a high fat diet also increased body weight and fat mass but did not significantly affect glucose and lipid homeostasis [64], which is similar to phenotype of the diet-induced obesity in FABP4 heterozygous knockout mice [14] and indicates that residual FABP4 protein sustains some elements of its function in metabolic control. 

In human and mouse monocyte cell lines, FABP4 expression is induced during differentiation from monocytes and by treatment with phorbol 12-myristate 13-acetate, lipopolysaccharide (LPS), PPAR*γ* agonists, and oxidized low-density lipoprotein (ox-LDL) [11, 34–37]. FABP4 expression in macrophages was also elevated by advanced glycation end products (AGE) via engagement of the receptor for AGE (RAGE) [38]. Conversely, a cholesterol-lowering statin, atorvastatin, has been shown to suppress FABP4 expression in macrophages *in vitro* [39]. It has also been reported that metformin, an antidiabetic drug, inhibits forkhead box protein O1- (FOXO1-) mediated transcription of FABP4, leading to reduced lipid accumulation in macrophages [40]. 

In macrophages, FABP4 modulates cholesterol ester accumulation and foam cell formation via inhibition of the PPAR*γ*-liver X receptor *α* (LXR*α*)-ATP-binding cassette A1 (ABCA1) pathway and induces inflammatory responses through activation of the IKK-NF-*κ*B and JNK-AP-1 pathways [41, 42]. Deficiency of FABP4 protected against atherosclerosis in apolipoprotein E- (ApoE-) deficient mice with or without high-cholesterol-containing western diets [11, 16]. Bone marrow transplantation studies demonstrated that the protective effect of FABP4 deficiency on atherosclerosis is predominantly related to actions in macrophages rather than in adipocytes [11]. FABP4 in dendritic cells has been shown to regulate the IKK-NF-*κ*B pathway and T-cell priming [12], which might contribute to the development of atherosclerosis since there is clear evidence for the involvement of both dendritic and T cells in the pathogenesis of atherosclerosis [65]. Involvement of FABP4 in atherosclerosis has also been indicated by clinical studies. In human endarterectomy samples of carotid stenosis, expression of FABP4 by macrophages was increased in unstable carotid plaques [66].

### 3.2. FABP5 (E-FABP/mal1)

Transgenic mice with adipose tissue-specific overexpression of FABP5 exhibited enhanced basal and hormone-stimulated lipolysis and a decrease in insulin sensitivity in a high-fat diet model [17, 58]. Deletion of FABP5 resulted in a mild increase in systemic insulin sensitivity in genetic and dietary obesity mouse models [17]. Adipocytes in FABP5-deficient mice showed an increased capacity for insulin-dependent glucose transport. Except for increased FABP3 (H-FABP) in the liver [67], there was no compensatory increase in the expression of FABPs in tissues including adipose tissue in FABP5-deficient mice [17]. Interestingly, feeding a western-type high-cholesterol diet increased the expression of FABP5, but not that of FABP1 (L-FABP), in liver parenchymal cells of atherosclerotic LDL-receptor- (LDLR-) deficient mice together with an increase in plasma levels of atherogenic lipoproteins, VLDL and LDL [61]. These observations indicate a specific role of FABP5 in atherogenesis.

FABP5 expression in macrophages was increased by treatment with Toll-like receptor (TLR) agonists: LPS, a TLR4 agonist, and zymosan, a fungal product that activates TLR2 [59]. A recent study showed that macrophage FABP5 deficiency suppressed atherosclerosis in LDLR-deficient mice on a western-style diet through a reduction of the expression of inflammatory genes, cyclooxygenase-2 and interleukin 6, and macrophage recruitment in atherosclerotic lesions due to decreased CC chemokine receptor 2 expression [60].

### 3.3. Combined Deficiency of FABP4 and FABP5

Mice with combined deficiency of FABP4 and FABP5 (*Fabp4^−/−^Fabp5^−/−^*) on a high-fat diet or in a genetic obesity model exhibit remarkably improved insulin resistance and protection against type 2 diabetes and fatty liver disease more than did FABP4- or FABP5-deficient mice [18, 19]. Furthermore, *Fabp4^−/−^Fabp5^−/−^* mice intercrossed into an ApoE-deficient atherosclerosis model developed dramatically less atherosclerosis than that in FABP4-deficient or wild-type mice on the same background [20]. Interestingly, *Fabp4^−/−^Fabp5^−/−^Apoe^−/−^*  mice on a western-type hypercholesterolemic diet also had a significantly higher survival rate than that of *Apoe^−/−^*  mice, presumably due to better plaque stability and good overall metabolic health [20].

**Figure 1:**
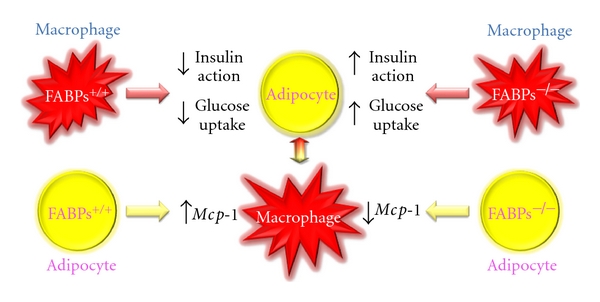
Interaction of adipocytes and macrophages. FABPs, FABP4, and FABP5, in adipocytes and macrophages, are critical for regulating inflammatory and metabolic responses in each type of cells and also interaction of the two types of cells.

It has recently been suggested that macrophage infiltration and accumulation in adipose tissue is an important feature of metaflammation triggered by obesity [68, 69]. Although the impact of FABP4/FABP5 on atherosclerosis was shown to be mainly due to actions in macrophages [11, 60], cell-based coculture experiments with adipocytes and macrophages and bone marrow transplantation using wild-type and *Fabp4^−/−^Fabp5^−/−^* mice showed that FABP actions in both adipocytes and macrophages have distinct roles in modulation of insulin sensitivity through inflammatory and metabolic responses as shown in Figure 1 [21]. In this setting, the predominant action was related to adipocyte FABPs with a more modest contribution from macrophages.

## 4. Therapeutic Target for Diabetes and Atherosclerosis

**Figure 2:**
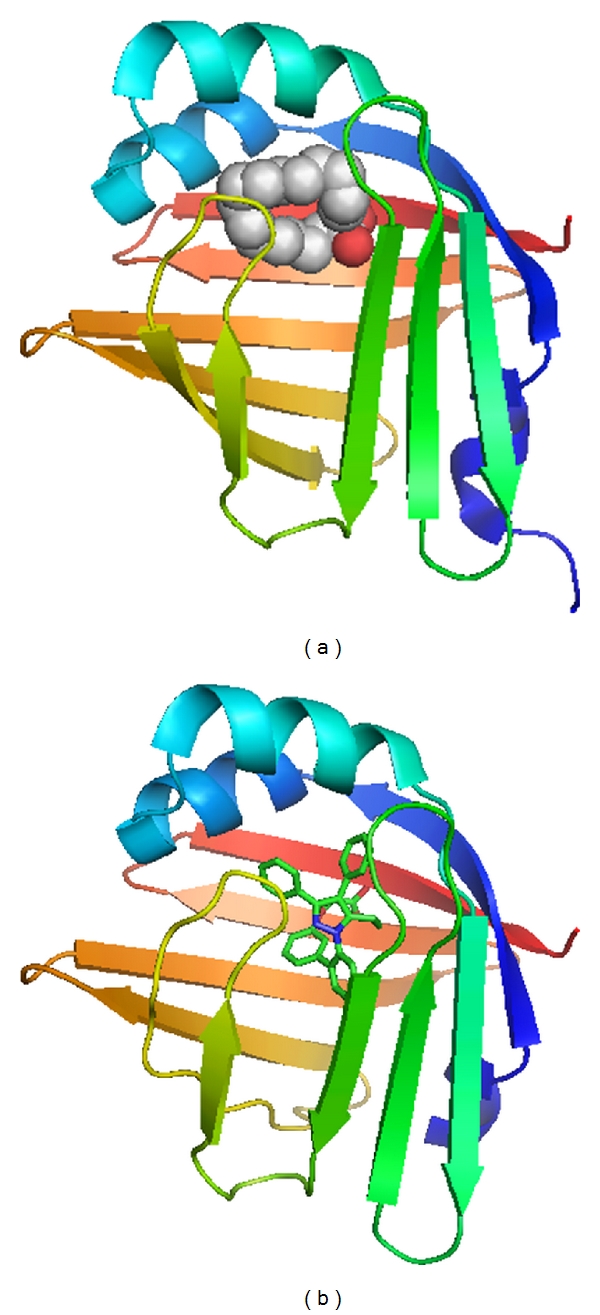
FABP4 bound with a fatty acid or a small molecule inhibitor. (a) Human FABP4 binds to an endogenous fatty acid, palmitic acid, as a twisted U-shaped entity in the binding pocket (PDB code: 2hnx). (b) Human FABP4 crystallized in complex with BMS309403, a synthetic FABP4 inhibitor, is shown (PDB code: 2nnq). The molecule occupies the internal binding pocket of FABP4 and competitively inhibits binding of endogenous fatty acids. The figures were created using PyMOL.

Since FABP4 and FABP5 act at the interface of metabolic and inflammatory pathways and play a significant role in the development of insulin resistance, type 2 diabetes, and atherosclerosis, it is expected that modification of the function of these FABPs may provide a new class of multi-indication therapeutic agents. In fact, several series of FABP4 inhibitors have recently been identified [70–75]. We previously demonstrated that chemical inhibition of FABP4 could be a therapeutic strategy against insulin resistance, diabetes mellitus, fatty liver disease, and atherosclerosis in experimental models using one of the specific FABP4 inhibitors, BMS309403 [33]. This compound is an orally active small molecule and interacts with the fatty acid-binding pocket within the interior of FABP4 to inhibit binding of endogenous fatty acids [7, 33, 72] (Figure 2). X-ray crystallographic studies identified the specific interactions of BMS309403 with key residues, such as Ser53, Arg106, Arg126, and Tyr128, within the fatty-acid-binding pocket as the basis of its high *in vitro* binding affinity and selectivity for FABP4 over other FABPs [72].

The FABP4 inhibitor, BMS309403, improved glucose metabolism and enhanced insulin sensitivity in both dietary (high fat-fed) and genetic (*ob/ob*) mouse models of obesity and diabetes [33]. Involvement of FABP4 inhibition in those beneficial effects was confirmed *in vivo* using wild-type and *Fabp4^−/−^Fabp5^−/−^* mice. Although *Fabp4^−/−^* mice were not protected against fatty liver disease, inhibition of FABP4 suppressed fatty liver infiltration, similar to the liver phenotype of *Fabp4^−/−^Fabp5^−/−^* mice. One possible explanation for the different effects between genetic deficiency of FABP4 and chemical inhibition of FABP4 is that there was no compensatory increase in FABPs in the adipose tissue of FABP4-inhibitor-treated mice. Furthermore, the FABP4 inhibitor markedly reduced the extent of atherosclerotic lesions in ApoE-deficient mice [33]. Cell-based studies showed that BMS309403 reduced macrophage foam cell formation with decreased cholesterol ester accumulation, increased cholesterol efflux, and decreased production of several inflammatory mediators in a target tissue-specific manner [33]. 

In high fat-diet-induced obesity models beginning at 4 weeks of age, treatment with the FABP4 inhibitor for 4 weeks improved insulin sensitivity in 24-week-old mice [33], which had severe macrophage infiltration in adipose tissue, but not in 20-week-old mice, which had much less macrophage accumulation in adipose tissue (Furuhashi M and Hotamisligil GS. unpublished data 2007). Recently, a similar pattern was also found in another study in which a different inhibitor was not effective in increasing insulin sensitivity [75]. It is difficult to completely inhibit whole FABP4 in adipocytes because the amounts of FABP4 in adipose tissue and adipocytes are very large [8], and these observations therefore raise the possibility that small molecules developed so far against FABP4 may be more effective in macrophages and hence their effects *in vivo* may be related to the extent of macrophage involvement with the disease process at the stage that these molecules are tested. Undoubtedly, future studies and alternative strategies to modulate FABP action, alone or in combination, in disease models should address these outstanding issues. Further studies are also needed to determine whether chemical or other modes of inhibition of FABP4 can be safely used in humans and to demonstrate their efficacy for metabolic diseases.

## 5. Ectopic Expression of FABP4

There is accumulating evidence to indicate that FABP4 is expressed in several cells other than adipocytes and macrophages under both special and physiological conditions (Table 1). For example, FABP4 expression was observed in endothelial cells of capillaries and small veins in several mouse and human tissues, including the heart and kidney [43]. FABP4 was significantly induced by treatment with vascular endothelial growth factor-A (VEGF-A) via VEGF-receptor-2 (VEGFR2) and by treatment with basic fibroblast growth factor (bFGF) in endothelial cells [43]. Conversely, knockdown of FABP4 in endothelial cells reduced proliferation both under baseline conditions and in response to VEGF-A and bFGF, suggesting that FABP4 is a target of the VEGF-A/VEGFR2 pathway and a positive regulator of cell proliferation in endothelial cells. 

Interesting observations have been reported for roles of FABP4 in vascular injury. FABP4 was markedly upregulated in regenerated endothelial cells obtained after endothelial balloon denudation *in vivo* [44]. In human aortic endothelial cells, intermittent hypoxia increased FABP4 expression [45]. Anigiopoietin-1, which participates in blood vessel stabilization and remodeling together with angiopoietin-2, inhibited FOXO1-mediated expression of genes including FABP4 in endothelial cells [46]. FABP4 was expressed in the aortic endothelium of 12-week-old ApoE-deficient mice showing endothelial dysfunction, whereas FABP4 was not detected at the aortic endothelium in 8-week-old ApoE-deficient mice or in wild-type mice [47]. Chronic administration of BMS309403, a small molecule FABP4 inhibitor, significantly improved endothelial dysfunction in ApoE-deficient mice [47]. Notably, recent studies have shown possible involvement of FABP4/FABP5 in senescence of endothelial cells [48, 49]. These observations support the notion that pathological induction, but not physiological expression, of FABP4 in the endothelium significantly contributes to pathogenesis of atherosclerosis and other types of vascular injury.

Evidence is also accumulating as for involvement of FABP4 in respiratory diseases. Recently, FABP4 has been reported to be detected in lungs and bronchoalveolar samples from patients with bronchopulmonary dysplasia (BPD) [50]. Density of FABP4-positive endothelial cells was increased in peribronchial blood vessels, and FABP4 was also localized in a subset of macrophages in lung tissues. Several studies using lung lavage cells suggested that FABP4 gene expression is responsible for pathogenesis of sarcoidosis [51]. It is notable that expression of FABP4 in human bronchial epithelial cells is under regulation of cytokines. FABP4 expression in bronchial epithelial cells was enhanced by the Th2 cytokines IL-4 and IL-13, which are involved in development of asthma, and was suppressed by the Th1 cytokine interferon *γ* [13]. Interestingly, FABP4-deficient mice were protected from airway inflammation independently of bone marrow-derived elements, indicating possible protection against asthma through FABP action in stromal cells [13]. However, it should be noted that there are possible differences in response of FABP4 to stimuli depending on cell types. FABP4 expression in bronchial epithelial cells was significantly lower than that in adipocytes and macrophages, even after stimulation. In contrast to its effects in adipocytes and macrophages, PPAR*γ* agonists could not induce FABP4 expression in bronchial epithelial cells. Such tissue-specific roles and response of FABP4 need to be taken into account for FABP4 modulating therapy.

In atretic antral follicles of the mouse ovary, FABP4 was detected in apoptotic granulosa cells [52], suggesting a possible relevance to polycystic ovary syndrome (PCOS), which often coexists with insulin resistance. Interestingly, association between FABP4 gene polymorphisms and the development of PCOS has been reported [53]. Additionally, dexamethasone treatment induced FABP4 in mouse spleen and in cultured T lymphocytes, and its distinct nuclear localization occurred with the dexamethasone-induced apoptosis process [54]. 

FABP4 expression was also detected in lipoblasts in lipoblastoma and liposarcoma but not in other benign adipose tissue or malignant connective tissue or in epithelial tumors [56]. Moreover, FABP4 expression has been linked to human urothelial carcinomas [57]. The significance of these associations remains to be elucidated but points to potential utility of FABP-based strategies to explore metabolic mechanisms related to tumorigenesis and related therapeutic possibilities.

## 6. Secretion and Circulating Concentrations of FABPs

In recent years, numerous studies have shown the presence of FABPs in circulation. Since these cytoplasmic proteins lack a secretory signal sequence, the presence of FABPs in serum is considered to be a biochemical marker of tissue injury in related cells that produce FABP proteins: FABP3 (H-FABP) for acute myocardial infarction and ongoing myocardial damage in heart failure, FABP7 (B-FABP) for brain injury, and FABP2 (I-FABP) for intestinal damage [76–78]. It has recently been reported that FABP4 is detected in serum and cultured adipocyte supernatants [79] and that the serum concentration of FABP4 is associated with obesity, type 2 diabetes, and cardiovascular diseases [79–82]. Similar findings have also been reported for FABP5 [83, 84]. Proteomics analysis using differentiated THP-1 macrophages revealed the presence of FABP4 and FABP5 in cell supernatants derived from macrophages [85]. However, the mechanisms and biological correlates of extracellular FABP4 and FABP5 remain unknown.

Serum levels of FABP4 were significantly increased in overweight and obese subjects compared to the level in lean controls and were positively correlated with waist circumference, blood pressure, and insulin resistance [79]. Similar to FABP4, circulating FABP5 levels were detected at the level of about one tenth or less of FABP4 concentrations and were associated with metabolic syndrome components [83, 84]. High serum levels of FABP4 at baseline independently predicted the development of metabolic syndrome during a 5-year follow-up period in a Chinese population [80]. A 10-year prospective study showed that high FABP4 concentration at baseline was a biomarker predicting development of type 2 diabetes, which was independent of obesity and insulin resistance [81]. Furthermore, it has also been reported that serum FABP4 levels are positively correlated with carotid intima-media thickness as an index of atherosclerosis [82]. These findings support the notion that FABP4 is a biomarker of ongoing atherosclerosis. Interestingly, serum levels of FABP4 could also represent noncardiovascular pathologic processes as well. A recent study has shown that FABP4 levels could be a novel and obesity-independent prognostic factor in patients with breast cancer [86].

Several drugs have been reported to modify FABP4 levels in blood. Atorvastatin, a HMG-CoA reductase inhibitor, and olmesartan, an angiotensin II receptor blocker, reduced circulating FABP4 levels [87, 88], whereas pioglitazone, an insulin-sensitizing thiazolidinedione (a PPAR*γ* agonist), increased FABP4 concentrations [89], which could be explained through direct activation of PPAR*γ* since the PPAR response element is present in the FABP4 gene promoter [90]. As general information for circulating FABPs, the concentrations of FABPs are influenced by renal clearance [91–93], and it might be necessary to evaluate the role of renal dysfunction in regulation of FABP level. Future studies should provide further insights into these phenomenon and how they contribute to disease progression in related FABP isoforms.

## 7. Lipokine

Meticulous lipidomic analyses using several samples including adipose tissue, liver, skeletal muscle, and blood from *Fabp4^−/−^Fabp5^−/−^* and wild-type mice showed markedly increased *de novo* lipogenesis in adipose tissue resulting from activation/induction of fatty acid synthase (FAS) and stearoyl*-*CoA desaturase*-*1 (SCD-1) [94]. Consequently, an unsaturated free fatty acid, palmitoleate (C16:1n7), was identified as an adipose tissue-derived lipid hormone, referred to as “lipokine,” that strongly suppresses hepatosteatosis and stimulates glucose transport in skeletal muscle [94]. That study revealed a lipid-mediated endocrine network of tissues/organs, in which adipose tissue uses lipokines such as palmitoleate to communicate with distant organs, regulating systemic metabolic homeostasis. Absence of FABP4 in macrophages also resulted in an activation of *de novo *lipogenesis pathways particularly through LXR*α*-mediated activation of SCD-1 [22]. This enhanced lipogenesis induced production of bioactive lipids including palmitoleate and resistance to ER stress. These changes also translate into protection against atherosclerosis in mouse models [22]. Conversely, unsaturated fatty acids including palmitoleate repressed basal and LPS-induced FABP4 expression in macrophages via the modulation of histone deacetylation [95].

After results of animal studies on a lipokine were reported [94], palmitoleate in humans was examined in several studies in the context of metabolic disease, particularly in determining the risk for insulin resistance and type 2 diabetes. In a study that recruited 100 Caucasian subjects, circulating palmitoleate was positively correlated with insulin sensitivity assessed by euglycemic-hyperinsulinemic clamp studies, independent of age, gender, and adiposity [96]. Another study using 3630 subjects in the US showed that high concentrations of circulating *cis* isomer palmitoleate, which is primarily produced by the liver in humans, were associated with adiposity, carbohydrate consumption, and alcohol use [97]. However, the associations between circulating *cis* palmitoleate and metabolic risk factors were complex, perhaps related to divergent lifestyle determinants or tissue sources of endogenous palmitoleate synthesis from liver and adipose tissue: high fat- and carbohydrate-containing diet and fatty liver would confound or modify the ability to detect its metabolic effects [97]. Interestingly, it has recently been reported that circulating *trans* isomer of palmitoleate, an exogenous source of C16:1n7, is associated with markedly lower insulin resistance, higher HDL-cholesterol level, and lower incidence of diabetes, suggesting metabolic benefits of dairy product consumption [98]. Since this isoform is not related to endogenous production, the relation to reduced metabolic disease points to possibilities of the utilization of the *trans* isomer of palmitoleate as a potential strategy for intervention in human diseases.

## 8. Concluding Remarks

FABPs, especially FABP4 and FABP5, play significant roles in the regulation of glucose and lipid metabolism linked to inflammatory and metabolic processes through modulating critical lipid-sensitive pathways in target cells, adipocytes, and macrophages. There was no compromised phenotype of FABP4- or FABP5-deficient mice under normal physiologic conditions [14, 17]. However, the mice in the context of dietary or genetic obesity were protected from systemic pathologic stresses such as metaflammation, suggesting that the adipocyte/macrophage FABP genes may represent an example of the “thrifty” gene hypothesis [99]. FABPs have been evolutionarily preserved from invertebrates (lower eukaryotes) to vertebrates including humans [100], indicating that a close and conserved link between inflammatory and metabolic responses underlies the conservation of FABP function. The presence of these FABPs may have been beneficial for ensuring a strong macrophage immune response under pressure with pathogens or for maintaining adipose tissue energy stores as part of the “thrifty” phenotype to survive in famine. Under contemporary life-style accompanied by excessive caloric intake and decreased energy expenditure, presence or induction of adipocyte/macrophage FABPs may be rather disadvantageous for maintaining inflammatory or metabolic homeostasis. FABPs appear to be responsible for the development of obesity, diabetes, dyslipidemia, and atherosclerosis, and targeting the adipocyte/macrophage FABPs, particularly FABP4, offers highly attractive therapeutic opportunities for intervening metabolic derangements as an evolutionary bottleneck in humans. Much work is still needed to elucidate the precise biological functions of different forms of FABPs and to establish strategies to target these proteins for therapeutic purposes.

## Figures and Tables

**Table 1:** Features of FABP4 and FABP5 in metaflammation and related diseases.

	Expression	Regulation and function	Connection to diseases	Reference
FABP4	Adipocyte	Induction by fatty acids, PPAR*γ* agonists, dexamethazone, and insulin		[23–27]
		Lipolysis (interaction with HSL)		[28–30]
		Regulation of insulin secretion during lipolysis		[29]
		Fatty acid sensor (interaction with JAK2)		[31]
		Regulation of lipid metabolism and differentiation (interaction with PTEN)		[32]
		Protection from insulin resistance and diabetes in deficient mice	Insulin resistance, diabetes	[14, 15, 18, 19, 21]
		Protection from insulin resistance and diabetes by a FABP4 inhibitor	Insulin resistance, diabetes	[33]
	Macrophage	Induction by PMA, LPS, PPAR*γ* agonists, ox-LDL, and AGE/RAGE		[11, 34–38]
		Reduction by atorvastatin and metformin		[39, 40]
		Activation of IKK-NF-*κ*B pathway		[41]
		Activation of JNK-AP-1 pathway		[42]
		Inhibition of PPAR*γ*-LXR*α*-ABCA1 pathway		[41]
		FOXO1-mediated transcription		[40]
		Association with ER stress		[22]
		Protection from insulin resistance and diabetes in double-deficient mice*	Insulin resistance, diabetes	[21]
		Protection from atherosclerosis in deficient mice	Atherosclerosis	[11, 16, 20]
		Protection from insulin resistance and atherosclerosis by a FABP4 inhibitor	Insulin resistance, atherosclerosis	[33]
	Dendritic cell	Activation of IKK-NF-*κ*B pathway		[12]
		T-cell priming		[12]
	Endothelial cell	Expression in capillary and small vein but not in artery		[43]
		Regulation by VEGF-A/VEGFR2 and bFGF		[43]
		Induction in regenerated endothelial cells after balloon denudation of artery		[44]
		Induction by intermittent hypoxia		[45]
		FOXO1-mediated transcription inhibited by angiopoietin-1		[46]
		Expression in aortic endothelium of old ApoE-deficient mice		[47]
		Improvement of dysfunction in aortic endothelium by a FABP4 inhibitor	Endothelial dysfunction	[47]
		Association with oxidative stress and activation of NF-*κ*B and P53 pathways	Cellular senescence	[48, 49]
	Bronchial epithelial cell	Induction by Th2 cytokines IL-4 and IL-13		[13]
		Suppression by Th1 cytokine interferon *γ*		[13]
		Noninduction by PPAR*γ* agonists		[13]
		Protection from asthma in deficient mice	Asthma	[13]
	Lung	Detection in lung lavage cells obtained from patients	Bronchopulmonary dysplasia	[50]
		Detection in lung lavage cells obtained from patients	Sarcoidosis	[51]
	Ovary	Expression in granulosa cells inside atretic antral follicles		[52]
		Association with FABP4 gene polymorphisms	Polycystic ovary syndrome	[53]
	Spleen	Induction by dexamethazone		[54]
	T cell	Induction by dexamethazone		[54]
	Keratinocyte	Induction in PTEN-deficient keratinocytes		[55]
	Tumor	Detection in tumor	Lipoblastoma, liposarcoma	[56]
		Detection in tumor	Urothelial carcinoma	[57]
FABP5	Adipocyte	Lipolysis		[58]
		Protection from insulin resistance and diabetes in deficient mice	Insulin resistance, diabetes	[17–19, 21]
		Induction of insulin resistance in adipose-specific transgenic mice	Insulin resistance, diabetes	[17]
	Macrophage	Regulation by TLR agonists: LPS (TLR4) and zymosan (TLR2)		[59]
		Induction of inflammatory genes, COX2 and IL-6		[60]
		Protection from insulin resistance and diabetes in double-deficient mice*	Insulin resistance, diabetes	[21]
		Protection from atherosclerosis in deficient mice	Atherosclerosis	[20, 60]
	Liver	Induction by a high-cholesterol diet feeding in LDL-receptor-deficient mice		[61]
	Others	Expression in skin, dendritic cell, tongue, mammary gland, brain, intestine, kidney, lung, heart, skeletal muscle, testis, retina, lens, and spleen		[7]

ABCA1: ATP-binding cassette A1; AGE: advanced glycation end products; AP-1: activating protein-1; ApoE: apolipoprotein E; bFGF: basic fibroblast growth factor; COX2: cyclooxygenase-2; ER: endoplasmic reticulum; FOXO1: forkhead box protein O1; HSL: hormone-sensitive lipase; IKK: inhibitor of nuclear kappa B kinase; IL: interleukin; JAK2: Janus kinase 2; JNK: c-Jun N-terminal kinase; LDL: low-density lipoprotein; LPS: lipopolysaccharide; LXR: liver X receptor; NF-*κ*B: nuclear factor-kappa B; ox-LDL: oxidized LDL; PMA: phorbol 12-myristate 13-acetate; PPAR: peroxisome proliferator-activated receptor; PTEN: phosphatase and tensin homolog on chromosome 10; RAGE: receptor for AGE; TLR: Toll-like receptor; VEGF-A: vascular endothelial growth factor-A; VEGFR2: VEGF-receptor-2.

*FABP4^−/−^FABP5^−/−^ mice.
